# Cultivating a rural nursing workforce: the impact of rural vs. urban education on nursing students’ intentions for rural practice in Nepal

**DOI:** 10.1186/s12912-026-04722-9

**Published:** 2026-05-08

**Authors:** Animesh Ghimire, Mamata Sharma Neupane

**Affiliations:** 1 Sustainable Environment Foundation Nepal, Baneshwor-31, Kathmandu, Nepal; 2 School of Nursing and School of Public Health, Chitwan Medical College, Bharatpur-5, Kailashnagar, Chitwan Nepal

**Keywords:** Rural health workforce, Nursing education, Rural immersion, Nepal, Theory of planned behaviour, Structural intervention, Gender

## Abstract

**Background:**

Rural–urban maldistribution of health professionals continues to widen health inequities, particularly in low- and middle-income countries (LMICs). In Nepal, nurses are central to frontline care in remote communities, yet “urban pull” forces and structural constraints undermine rural recruitment and retention. This study examined how training at a rural-embedded institution—Karnali Academy of Health Sciences (KAHS)—may shape final-year nursing students’ intentions for rural/remote practice and the barriers they anticipate.

**Methods:**

We used a two-site qualitative descriptive design with an exploratory orientation. Semi-structured interviews were conducted with 22 final-year female nursing students: 11 from an urban tertiary institution and 11 from KAHS. A codebook-style applied thematic analysis was undertaken. Ajzen’s Theory of Planned Behaviour and a structural intervention lens guided interpretation of how institutional context may influence beliefs underpinning rural-practice intentions.

**Results:**

Five themes captured students’ accounts: (1) rural immersion and emerging rural commitment; (2) experiential learning beyond the books; (3) the relational pull of community-centred care; (4) perceived urban–rural inequities and curriculum gaps; and (5) gendered and professional barriers to rural practice. Most KAHS participants—including several of urban origin—described strong intentions to begin their careers in rural/remote settings, whereas most urban-site participants anticipated urban employment. However, anticipated constraints—especially safety concerns, social isolation, professional/academic isolation, and limited career development pathways—were repeatedly described as factors that could weaken the feasibility and sustainability of rural practice.

**Conclusion:**

Rural-embedded education may strengthen intentions to serve in rural areas by reshaping how students evaluate rural work (attitudes), social expectations (subjective norms), and the feasibility of rural service (perceived behavioural control). Yet intention formation occurs alongside persistent gendered and structural barriers. Longitudinal research is needed to track post-graduation trajectories and to identify multi-component strategies that translate rural intention into durable rural retention.

**Clinical trial number:**

Not applicable.

## Background

The maldistribution of healthcare professionals between urban and rural areas is a pervasive global challenge that affects both high-income countries (HICs) and low- and middle-income countries (LMICs). Yet, in LMICs, the consequences of this maldistribution are particularly severe [1–3]. Global policy guidance further highlights that education strategies—such as targeted recruitment from underserved communities, sustained rural clinical exposure, and training institutions located in rural/remote regions—are among the core levers for improving rural workforce supply and retention [4]. Nepal, a lower-middle-income country with a gross national income (GNI) per capita of USD 1,470 [5], grapples with formidable barriers to healthcare delivery, especially in its rural regions, which are home to nearly 33% of the country’s population [6]. Although this rural demographic bears the greatest burden of health inequities, the concentration of medical facilities, resources, and skilled professionals in urban centres has created a persistent “urban pull” [7]. Advanced infrastructure, career prospects, and better living conditions draw healthcare workers away from underserved areas [8], leaving rural communities with insufficient personnel, scarce medical supplies, and limited access to essential services [9–11].

These inequities are further magnified by Nepal’s rugged topography and its socioeconomic realities. Rural populations bear disproportionate mortality risks, restricted access to maternal and child health services, and limited capacity to manage chronic conditions [12–14]. In particular, Karnali Province stands out for the severe challenges it faces. Karnali Province is situated in the north-western part of Nepal and borders the Tibet Autonomous Region of China to the north; it adjoins Gandaki Province to the east, Lumbini Province to the south, and Sudurpashchim Province to the west [15, 16]. Bagmati Province—home to Nepal’s capital city, Kathmandu—lies further east [17]. According to the Nepalese Government Poverty Index, 39.5% of Karnali’s population faces multidimensional poverty—the highest among all provinces [18]. Such hardship is reflected in health indicators, including maternal and infant mortality and underutilization of skilled birth attendants [19, 20]. These intersecting factors perpetuate a cycle in which communities with the greatest need are the least served, undermining progress toward equitable healthcare outcomes [21, 22].

While the maldistribution of healthcare professionals poses a global challenge, it is crucial to recognise that nurses are the cornerstone of healthcare delivery worldwide. Comprising over 60% of the global health workforce, an estimated 29 million nurses provide the vast majority of direct patient care across diverse settings [23, 24]. Evidence suggests that nurse density is strongly correlated with crucial health outcomes, including reduced mortality rates and increased life expectancy [25, 26]. However, it is noteworthy that the nursing profession exhibits a significant gender disparity, with women constituting 67% of the global health and social care workforce [24]. This trend is also evident in the Nepalese nursing workforce, which is exclusively female [27]. Hence, in the context of the rural healthcare workforce, this gender dynamic becomes particularly salient. Longstanding sociocultural norms—encompassing traditional gender roles and societal expectations—can impede women’s willingness to work in remote areas [28–30]. Therefore, to augment the nursing labor workforce, Nepal implements a dual-track nursing education: a four-year Bachelor of Science in Nursing (BSc) following the completion of Year 12 of secondary education and a three-year Bachelor of Nursing Science (BNS) program for those with diploma level—Proficiency Certificate in Nursing (PCL Nursing) [31, 32]. Despite these efforts, maldistribution persists, underscoring the need to examine how educational settings and institutional structures influence nurses’ career decisions, particularly in rural contexts.

One promising avenue to address workforce shortages is structural intervention—a system-level initiative aimed at changing the social, economic, or institutional environment to achieve better health workforce outcomes [33]. In Nepal, an example of such an intervention is the establishment of the Karnali Academy of Health Sciences (KAHS) in the remote Karnali Province. Created in 2011 by an Act of Parliament to enhance healthcare through education, research, and community engagement [34, 35], KAHS is the only stand-alone tertiary institution of its kind in the country [36]. In line with its “stand-alone” mandate, the KAHS Act specifies that the Academy does not provide affiliation to other educational institutions and formally anchors training, service delivery, and research within Nepal’s designated “backward areas,” with its central office in Jumla [37]. Importantly, the Act also embeds a rural pipeline logic by requiring reservation of at least 45% of admissions up to Bachelor’s level for students permanently residing in designated backward areas, providing at least 20% full scholarships to students classified as poor, and linking scholarship/reservation to a compulsory period of service in remote areas [37].

KAHS’s nursing education model operationalises this structural intent through a community-oriented training approach and locally embedded practice opportunities. The School of Nursing and Midwifery, established in 2012, reports graduating multiple cohorts of certificate-level nurses and currently offers university-level nursing pathways (including BNS and BSc Nursing), with an explicitly community-based conceptual framework and emphasis on self-directed learning [36]. Alongside academic coursework, KAHS describes routine community engagement through health camps and outreach initiatives, positioning community-based teaching and learning as a core educational principle and aligning training with local cultural contexts and health practices [38]. Collectively, these institutional features (rural location, admissions/scholarship structures, community-based pedagogy, and embedded service opportunities) provide critical context for interpreting how training “in place” may shape students’ perceptions of rural practice.

To contrast this rural, legislatively mandated model with an urban tertiary setting, the present study compares KAHS with Chitwan Medical College (CMC), located in Bharatpur, Chitwan. CMC describes itself as a private medical college and teaching hospital established in 2006, reporting 19 + academic programs, 1,600 + staff members, and 30,000 + graduates across its health-sciences portfolio [39]. The School of Nursing at CMC boasts a highly qualified faculty and staff who support its diverse nursing programs. These programs encompass a range of specialisations and provide a unique training environment integrated within an urban tertiary-care setting [40]. This juxtaposition—between a rural, policy-anchored academy designed to serve underserved areas and an urban, resource-rich private medical college—enables a closer examination of how institutional context and educational immersion may influence nursing students’ intentions to practice in underserved regions.

Against this backdrop, the present study seeks to explore how final-year nursing students perceive this structural intervention and whether immersion in a rural academic setting influences their career intentions. By comparing the perspectives of students at KAHS—Nepal’s only stand-alone rural tertiary institution—with those of students at an urban tertiary institution (Chitwan Medical College), we aim to clarify whether a rural-focused training model fosters stronger intentions toward rural practice. Recognizing the formative influence of educational experiences on career trajectories, this research addresses the following question: In what ways, and to what extent, does immersion in a dedicated rural healthcare education setting (KAHS), as compared to an urban counterpart, influence final-year nursing students’ perceptions of—and commitment to—serving in underserved regions of Nepal, and which socio-structural factors mediate these outcomes? By illuminating these dynamics, we offer insights into the conditions under which future nurses might commit to rural practice, thereby informing more effective workforce-planning strategies and policy interventions that enhance equitable healthcare provision in remote and marginalised areas.

## Methods

### Theoretical framework

This study was guided by Ajzen’s Theory of Planned Behaviour (TPB) [41] and sensitised by structural intervention theory, which conceptualises system-level changes to social, economic, or institutional environments as mechanisms to reduce health inequities [33, 42]. The TPB suggests that behavioural intention, as the closest predictor of actual behaviour, is influenced by three key components: attitudes, which encompass behavioural beliefs regarding the anticipated consequences of the behaviour; subjective norms, reflecting normative beliefs about the expectations of significant others; and perceived behavioural control (PBC), which pertains to individuals’ beliefs about the factors that facilitate or impede their ability to perform the behaviour. Meta-analytic evidence indicates that TPB explains substantial variance in both intention and behaviour across contexts and behaviours [43]. Within TPB, PBC reflects perceived capability and controllability, including self-efficacy and the extent to which individuals perceive the behaviour as feasible given external constraints [44].

In this study, “intention for rural practice” referred to participants’ anticipated likelihood of accepting rural/remote nursing employment after graduation. Attitudes encompassed perceived benefits and trade-offs of rural work (e.g., professional meaning, workload, and resource constraints); subjective norms captured perceived expectations from family, peers, faculty, and communities; and PBC reflected perceived feasibility given living/working conditions, safety, supervision, and career-development opportunities. TPB was applied a priori to structure interview domains and later to support interpretation by mapping emergent themes to TPB constructs, while the structural intervention lens supported examination of how institutional context (KAHS vs. an urban institution) may shape the beliefs underpinning intention.

### Study design

This study employed a two-site qualitative descriptive design with an exploratory orientation to compare final-year nursing students’ perspectives across urban and rural educational settings. Qualitative description is suited to producing a comprehensive summary of experiences in participants’ everyday terms and staying close to the surface of words and events [45, 46], consistent with the study’s applied workforce-planning focus. The descriptive aim was to document students’ current perceptions, experiences, and intentions regarding rural practice. The exploratory orientation was used to examine how and why these intentions were shaped within contrasting institutional contexts—including structural investments in rural health education, personal background, and perceived opportunities and constraints—where evidence remains limited [47]. To ensure methodological coherence, thematic analysis was used as an analytic technique to organise patterns within these descriptive accounts, primarily at a semantic level, rather than to develop a new theory [48].

### Sample and setting

This study was conducted at two tertiary nursing education institutions in Nepal: Chitwan Medical College (CMC), Bharatpur, Bagmati Province, and the Karnali Academy of Health Sciences (KAHS), Jumla, Karnali Province [49]. These sites were selected to enable comparison of nursing students trained in contrasting educational and service-delivery environments (an urban tertiary-care setting vs. a rural/remote setting). Both institutions provide residential accommodation (hostel-based living), which is relevant to the “immersion” component of rural training.

Participants were recruited using purposive sampling to obtain information-rich accounts from students positioned to reflect on training experiences and imminent employment decisions [50]. Eligible participants were (1) enrolled in the final year of a nursing degree at either institution and (2) had completed at least 75% of required clinical placements. Recruitment intentionally sought maximum variation across characteristics likely to shape intentions for rural practice—such as geographic origin and prior rural exposure—because these contextual experiences can plausibly influence TPB-relevant beliefs (attitudes, subjective norms, and perceived behavioural control) regarding rural employment [41, 51]. At the rural site, participants were drawn from the Bachelor of Nursing Science (BNS) pathway (entry after Proficiency Certificate Level nursing), whereas the urban site comprised Bachelor of Science in Nursing (BSc Nursing) students (entry after Year 12), reflecting Nepal’s dual-track nursing education model [31, 32]. To broaden perspectives within each site and reduce the risk of recruiting only highly motivated students, snowball sampling was also used [52]. Sampling continued until the study team judged the dataset to have sufficient “information power” for the study aim [53].

### Data collection

Data were collected through individual semi-structured interviews conducted by the first author (AG) between July and September 2024. Interviews were held at a time and private location convenient to participants to support confidentiality and reduce peer influence. Each interview lasted approximately 60–90 min, was audio-recorded with written consent, and was supplemented by contemporaneous field notes capturing contextual details, non-verbal cues, and interviewer reflections. Participants could respond in English or Nepali. No prior relationship existed between the researchers and participants, and all participants were reminded that participation was voluntary and that they could withdraw at any time without consequence.

The interview guide (Table 1) was designed to elicit beliefs underpinning intentions for rural practice, guided by the Theory of Planned Behaviour (TPB) [41] and sensitised by structural intervention theory [33, 42]. In line with TPB belief-elicitation guidance [54], domains were informed by literature on rural workforce maldistribution and retention barriers [1, 21, 55–57], rural experiential/community-based education and rural practice intentions [58–61], rural healthcare access constraints in Nepal [9, 10, 62], and gendered barriers affecting female health workers [28, 63, 64]. To minimise social desirability bias and avoid implying that rural practice was the “preferred” response, questions were phrased neutrally and accompanied by balanced probes (advantages and disadvantages), indirect/third-person prompts, and requests for concrete examples—strategies consistent with recommendations for detecting and limiting socially desirable responding in qualitative interviews [65]. Ongoing debriefing within the study team during data collection was used to reflect on interview dynamics and refine probing while maintaining neutrality.

**Table 1:** Interview questions

Question number	Interview question
1	Can you tell me about your background and what motivated you to pursue a career in nursing? *(Probes: Where are you from originally? Did you grow up in a rural or urban area? What factors influenced your decision to study nursing?)*
2	How would you describe your experience in the nursing program so far, particularly your clinical placements? *(Probes: What kinds of placements have you had? Any rural/remote exposure? What stood out?)*
3	What are your perceptions of working as a nurse in a rural/remote area of Nepal after you graduate? *(Probes: What might be appealing? What might be challenging?)*
4	What factors are most important to you when deciding where to work after graduation? *(Probes: salary*,* living conditions*,* professional development*,* family expectations*,* safety*,* etc.)*
5	*(For students studying in Karnali)* In what ways, if any, has studying at KAHS influenced how you think about rural/remote practice? *(Probes: Has it influenced your intentions? In what ways—positive or negative?)*
6	*(For students studying in the urban setting)* How prepared do you feel your education has made you for working in rural/remote settings? Why? *(Probes: What prepared you well? What gaps remain?)*
7	What are your views on the availability and quality of healthcare services in rural Nepal? *(Probes: What do you see as key constraints and enabling factors for care delivery?)*
8	What policies, supports, or changes do you think would influence health workers’ decisions to take up—and remain in—rural/remote postings? *(Probes: incentives*,* supervision/mentorship*,* housing*,* transport*,* safety/security*,* career pathways*,* education reforms*,* etc.)*
9	As a female healthcare professional, what concerns (if any) do you anticipate regarding safety, social isolation, or work–life conditions in rural/remote practice? *(Probes: What kinds of supports would help?)*
10	How do you currently see your likelihood of working in a rural/remote setting after graduation, and why? *(Probes: What would make you more likely/less likely to do so?)*

### Data analysis

A codebook-oriented applied thematic analysis was undertaken, aligning with the principles of a qualitative descriptive methodology [48]. This analytic approach was selected to provide a structured, low-inference thematic summary while maintaining methodological coherence. First, all interviews were transcribed verbatim; where interviews were conducted in Nepali, transcripts were translated into English by AG and checked by MSN against the audio to support accuracy and meaning preservation [66]. Second, the research team familiarised themselves with the dataset through repeated reading and memoing. Third, initial codes were generated inductively, primarily at the semantic level [67]. Fourth, codes were organised into candidate categories and themes, which were then reviewed against the coded extracts and the full dataset to ensure coherence and distinctiveness. Fifth, themes were refined, defined, and named using descriptive, analytic-neutral labels. Sixth, the report was produced by selecting illustrative excerpts and finalising theme definitions. TPB constructs were used as sensitising concepts during interpretation (i.e., to consider how themes reflected attitudes, subjective norms, and perceived behavioural control), without displacing the inductive coding process [41]. To enhance analytic consistency, AG and MSN independently coded a subset of transcripts and reconciled coding differences through discussion, refining code definitions and decision rules in line with guidance on collaborative coding [68]. Analysis continued until no substantively new codes or theme refinements were identified, indicating adequate saturation for the study aims [69]. Table 2 summarises the analytic pathway from meaning units to themes.

**Table 2:** Data analysis process

Exemplar Meaning Unit	Code(s)	Category	Sub-theme	Theme
“My journey to Karnali was initially driven by pragmatism—it was a means to an end. Yet, immersion in the academy and community outreach profoundly altered my perspective. I’ve witnessed firsthand the impact we can make, and now I want to begin my career in a rural setting.” (Student Nurse 20, Urban origin, studying rurally)	Shift in perspective; personal transformation	Motivations for rural practice	Catalyst for new commitment	Theme 1: Rural immersion and emerging rural commitment
“I have always known the struggles of accessing quality healthcare in rural communities… Now, I know that I can make a great impact in rural settings after graduation.” (Student Nurse 5, Rural origin, studying rurally)	Recognition of local needs; renewed determination	Motivations for rural practice	Validation of rural focus	Theme 1: Rural immersion and emerging rural commitment
“Growing up in a rural area… it was about witnessing [diseases’] effects on the faces of the children we immunised… This was healthcare in its purest, most impactful form… It’s the tangible difference we make here that inspires my desire to serve this community after graduation.” (Student Nurse 9, Rural origin, studying rurally)	Emotional engagement; firsthand experience	Transition from theory to practice	Immersive learning reinforces commitment	Theme 2: Experiential learning in rural practice settings
“Mannequins and actors taught me skills, but it never felt fully real. Karnali challenges you not just clinically but also emotionally—by understanding patients’ stories and tailoring care. All participants in the study were female, reflecting the World Health Organisation’s workforce data that indicates the nursing workforce in Nepal is exclusively female. It cemented my resolve to work in a rural setting.” (Student Nurse 18, Urban origin, studying rurally)	Real-world complexities; emotional connection	Transition from theory to practice	Disparity between simulated and authentic practice	Theme 2: Experiential learning in rural practice settings
“We’re woven into the community’s fabric—sharing in everyday joys and challenges. That bond makes rural practice fulfilling in ways I never expected.” (Student Nurse 7, Rural origin, studying rurally)	Community integration; sense of belonging	Relational dimension of care	Shared purpose driving commitment	Theme 3: Relational and community-centred care
“City healthcare often feels like quick transactions—patients come and go. Here, we build trust and collaborate with families. It’s a model I’m not sure I’ll find in a big city.” (Student Nurse 14, Urban origin, studying rurally)	Trust-based care; contrast with urban “transactions”	Relational dimension of care	Community-centric approach vs. transactional care	Theme 3: Relational and community-centred care
“In Kathmandu… everything was on hand—medicines, specialists, technology. Karnali was a stark contrast… It made me question how to address such deep-rooted inequalities.” (Student Nurse 18, Urban origin, studying rurally)	Stark resource disparity; ethical questions	Urban-rural resource gap	Recognition of systemic inequities	Theme 4: Perceived urban–rural inequities and curriculum gaps
“Our curriculum predominantly focuses on urban health issues… I wish our education tackled real-life rural constraints more extensively.” (Student Nurse 4, Rural origin, studying in an urban setting)	Urban-centric training; underprepared for rural	Urban-rural resource gap	Curriculum mismatch	Theme 4: Perceived urban–rural inequities and curriculum gaps
“Rural areas come with extra vulnerabilities for women—harassment, law enforcement gaps, being away from family… It’s always assessing your own safety.” (Student Nurse 16, Urban origin, studying in an urban setting)	Security concerns; gender vulnerability	Intersection of gender & geography	Heightened safety risks for rural female nurses	Theme 5: Gendered and professional barriers to rural practice
“There’s a real sense of disconnection from peers if you’re posted far from home… It can take a toll on your well-being.” (Student Nurse 20, Urban origin, studying rurally)	Social isolation; limited support	Intersection of gender & geography	Emotional burden of rural practice	Theme 5: Gendered and professional barriers to rural practice

### Rigour and reflexivity

Trustworthiness was addressed using the criteria of credibility, dependability, confirmability, and transferability, with strategies aligned to a qualitative descriptive study using applied thematic analysis [70].

Credibility was supported through concurrent member checking during interviews: AG summarised key points at the close of each interview and invited participants to clarify, confirm, or correct interpretations. Credibility was also enhanced through data source triangulation by comparing accounts from students educated in rural and urban institutions, enabling examination of convergent and divergent perspectives across contexts.

Dependability was strengthened through consistent use of the semi-structured interview guide across participants and through systematic documentation of procedural decisions, including sampling, interview conduct, and analytic decisions. Confirmability was supported by maintaining an audit trail of analytic memos and decision logs that documented how interpretations were developed and refined, providing transparency into how findings were grounded in the dataset.

Reflexivity was treated as integral to rigour. The research was conducted by two authors (AG and MSN). AG (the interviewer) maintained reflexive notes to document assumptions, emotional responses, and potential influences during data collection and early interpretation. MSN provided a critical interpretive counterpoint by reviewing emerging interpretations and questioning alternative explanations, which supported a balanced, evidence-led interpretation. This iterative reflexive practice enhanced analytic sincerity and methodological coherence [71]. Finally, transferability was supported by providing detailed descriptions of the study context, participant characteristics, and institutional settings, enabling readers to judge applicability to other rural/remote training contexts and LMIC health workforce settings.

### Ethical consideration

Ethical clearance was obtained from the Nepal Health Research Council (approval number-444/2024) and the institutional review boards of Chitwan Medical College (CMC) and Karnali Academy of Health Sciences (KAHS). Written voluntary informed consent was obtained from each participant, who was assured of their right to withdraw from the study at any time.

## Results

### Participant’s characteristics

Twenty-two final-year nursing students participated in this study: 11 from the urban institution (Chitwan Medical College; Student Nurses 1–11) and 11 from the rural institution (Karnali Academy of Health Sciences; Student Nurses 12–22). All participants in the study were female, aligning with the World Health Organisation’s (WHO) data that suggests the nursing workforce in Nepal is exclusively female [72]. The two cohorts reflected Nepal’s dual-track nursing education pathways: students at KAHS were enrolled in the Bachelor of Nursing Science (BNS) programme (a 3-year programme following Proficiency Certificate Level nursing), whereas students at Chitwan Medical College were enrolled in the Bachelor of Science in Nursing (BSc Nursing) programme (a 4-year programme entered after Year 12 of secondary education).

Participants ranged in age from 22 to 32 years (M = 25.1, SD = 2.4). Students in the rural KAHS cohort were, on average, older (M = 26.8, SD = 2.5) than those in the urban cohort (M = 23.5, SD = 1.2), likely reflecting the additional time required to complete diploma-level nursing training before entry into the BNS programme.

Most participants (81.8%) originated from urban areas. Notably, 72.7% of students at KAHS were originally from urban areas, indicating that the rural institution attracted students beyond its rural-origin population. Overall, 31.8% of participants were married, and 45.5% reported prior rural exposure; this exposure was substantially more common among KAHS students (72.7%) than among the urban cohort (18.2%). Table 3 summarises key socio-demographic characteristics.

**Table 3:** Socio-demographic characteristics of participants (*N* = 22)

Characteristic	Category	Urban (CMC), *n* = 11	Rural (KAHS), *n* = 11	Total (*N* = 22)
Age (years)	Mean (SD)	23.5 (1.2)	26.8 (2.5)	25.1 (2.4)
	Range	22–26	23–32	22–32
Place of origin	Urban	10 (90.9%)	8 (72.7%)	18 (81.8%)
	Rural	1 (9.1%)	3 (27.3%)	4 (18.2%)
Marital status	Single	9 (81.8%)	6 (54.5%)	15 (68.2%)
	Married	2 (18.2%)	5 (45.5%)	7 (31.8%)
Prior rural exposure	Yes	2 (18.2%)	8 (72.7%)	10 (45.5%)
	No	9 (81.8%)	3 (27.3%)	12 (54.5%)

### Findings

#### Theme 1: Rural immersion and emerging rural commitment

Participants across diverse backgrounds described how enrolling in the Karnali Academy of Health Sciences (KAHS) reshaped their career goals. Instead of framing this as a causal “effect,” the findings presented below illustrate participants’ self-reported perceptions regarding how their studies and training at a rural institution—along with their involvement in community outreach—shaped their views on where to launch their careers. Initially, some students, especially those hailing from urban backgrounds, described a transition from scepticism or ambivalence to a clearer sense of purpose and a newfound openness to rural practice after engaging with KAHS’s community-oriented learning environment.

*My journey to Karnali was initially driven by pragmatism—it was a means to an end. Yet*,* immersion in this institution and community outreach profoundly altered my perspective. I’ve witnessed first-hand the impact we can make*,* and now I want to begin my career in a rural setting after graduation.* (Student Nurse 20, Urban origin, studying rurally)

Some students from rural backgrounds emphasised how KAHS’s presence validated their desire to serve underserved communities:

*I have always known the struggles of accessing quality healthcare in rural communities*,* and that is one of the reasons why I decided to become a nurse. Although I was motivated*,* I used to believe that to make a significant difference*,* one has to work in urban settings. The establishment of KAHS has truly changed my beliefs. KAHS has shown that rural healthcare can be a priority*,* and by creating such an institution in rural Nepal*,* they have shown us all that we matter too. Now*,* I know that I can make a great impact in rural settings after graduation.* (Student Nurse 5, Rural origin, studying rurally)

For others, encountering rural realities in the classroom and clinical settings prompted reconsideration of prior assumptions about “best practice” as strictly urban-based:

*In Kathmandu* [capital city], *advanced facilities seemed the pinnacle of care. KAHS opened my eyes to a more holistic approach that’s woven into the social fabric. It’s given me deeper fulfilment and a mission I never imagined.* (Student Nurse 15, Urban origin, studying rurally)

Participants reported that immersion in a rural academic setting, combined with active involvement in local communities, was associated with a shift in intentions to pursue nursing careers in rural and remote regions. This trend was particularly evident among students who initially held predominantly urban-centric expectations regarding their professional trajectories.

#### Theme 2: Beyond the books: Karnali’s transformative experience

Participants consistently noted that hands-on rural clinical placements and community-facing activities bridged the gap between theoretical knowledge and authentic patient care. They described learning “in place” as making clinical knowledge actionable by requiring flexible decision-making under constrained resources, culturally responsive communication, and close engagement with patients’ everyday realities. Direct community engagement was often cited as the catalyst for a renewed commitment to a future in underserved areas.

*Growing up in a rural area*,* I became acquainted with the healthcare challenges*,* but it was during my clinical rotations in Jumla* [one of the ten districts of the Karnali province] *that the realities truly resonated with me. It wasn’t just about learning disease names; it was about witnessing their effects on the faces of the children we immunised and feeling the gratitude in a mother’s eyes when we clarified her child’s ailment. This was healthcare in its purest*,* most impactful form. My grades reflect my commitment*,* but it’s the tangible difference we make here that inspires my desire to serve this community after graduation.* (Student Nurse 9, Rural origin, studying rurally)

Those from urban backgrounds contrasted simulation-based training in larger cities with the real-world complexities of rural practice, emphasising that “authentic” care required not only technical competence but also emotional attunement and relational work with patients and families:

*Mannequins and actors taught me skills*,* but it never felt fully real. Karnali challenges you not just clinically but also emotionally—by understanding patients’ stories and tailoring care. It cemented my resolve to work in a rural setting.* (Student Nurse 18, Urban origin, studying rurally)

However, the transformative impact of rural exposure was frequently described as conditional rather than uncomplicated, as students also acknowledged tensions related to long-term commitments. Several participants framed rural practice as simultaneously meaningful and demanding, describing an ongoing negotiation between professional purpose and anticipated personal and career-related trade-offs:

*My time here has been a revelation*,* challenging many of my assumptions about healthcare and my role within it. The limited resources and demanding conditions have undeniably honed my clinical skills and fostered a resilience I never knew I had. Yet it has also sparked some uncertainty. The decision to commit to a rural career path is no longer as straightforward as I once imagined. While I’m drawn to the tangible impact and profound connections inherent in rural practice*,* I’m also aware of the sacrifices I have to make […]. It’s a decision that requires careful consideration*,* weighing the personal fulfilment against the professional and personal challenges that come with it.* (Student Nurse 22, Urban origin, studying rurally)

#### Theme 3: The human connection: the relational pull of rural practice

Many participants foregrounded the relational character of rural healthcare, describing care as embedded within community life and sustained through continuity, reciprocity, and shared responsibility. They contrasted this with what some perceived as more episodic and transactional interactions in urban hospitals, where high patient turnover and time pressure can constrain relationship-building.

*We’re woven into the community’s fabric—sharing in everyday joys and challenges. That bond makes rural practice fulfilling in ways I never expected.* (Student Nurse 7, Rural origin, studying rurally)

For several students who had relocated from urban areas, rural practice was described as reorienting “good care” toward trust, familiarity, and collaborative engagement with families. Participants noted that sustained relationships with patients and households created opportunities to tailor health education, negotiate culturally appropriate care plans, and see the longer-term effects of nursing work.

*City healthcare often feels like quick transactions—patients come and go. Here*,* we build trust and collaborate with families. It’s a model I’m not sure I’ll find in a big city.* (Student Nurse 14, Urban origin, studying rurally)

Students who grew up in rural communities further described a sense of cultural and social resonance with patients, suggesting that shared language, norms, and everyday lived experience supported more nuanced communication and mutual understanding. In their accounts, rural nursing extended beyond clinical tasks to include advocacy, informal counselling, and community health education.

*Our work transcends the clinical setting*,* permeating every aspect of community life. We’re not just healthcare providers; we’re advocates*,* educators*,* and partners in a shared journey towards health. This integrated approach*,* where health is viewed as a collective endeavour*,* is what makes rural practice so meaningful to me. It aligns with my belief that true well-being is rooted in strong communities*,* and that is why I’m committed to serving my village after graduation.* (Student Nurse 10, Rural origin, studying rurally)

Some participants described that exposure to KAHS’s community-facing model prompted them to reconsider earlier aspirations for urban tertiary hospitals, reframing professional fulfilment as participation in a locally anchored, team-based approach to health.

*I was initially drawn to the prestige and fast-paced environment of urban hospitals. However*,* KAHS has opened my eyes to a different kind of professional fulfilment—one that comes from being part of a close-knit team where everyone*,* from community leaders to families*,* plays a role in health. I now realise that this collaborative spirit and sense of shared ownership are rare and valuable. It has redefined my understanding of what it means to be a healthcare professional and made me seriously reconsider my career path. I want to stay and contribute to this model of care.* (Student Nurse 19, Urban origin, studying rurally)

Overall, participants framed relational connection—trust, belonging, and shared purpose—as a meaningful feature of rural practice that shaped their perceived desirability of rural work and, for some, strengthened intentions to serve in underserved communities after graduation.

#### Theme 4: Unequal access: the urban–rural healthcare divide

Reflecting on their education and clinical experiences, participants described an urban–rural gradient in resources that they experienced as a practical constraint on delivering safe, timely care. They emphasised that disparities were not limited to equipment and medicines, but also included infrastructure reliability, referral pathways, transport and distance barriers, and access to clinical guidance—conditions they felt shaped both day-to-day practice and longer-term career trajectories.

*In Kathmandu* [capital city], *everything was on hand—medicines*,* specialists*,* technology. Karnali is a stark contrast. Basic supplies can be scarce*,* and patients travel long distances for simple care. It made me question how to address such deep-rooted inequalities.* (Student Nurse 18, Urban origin, studying rurally)

Participants, particularly those studying in urban settings, also highlighted a perceived misalignment between an urban-oriented curriculum and the realities of rural service delivery. They suggested that limited attention to rural constraints (e.g., scarcity of diagnostics, fewer specialist supports, and broader scope-of-practice demands) left them feeling underprepared for rural postings.

*Our curriculum predominantly focuses on urban health issues. Rural health gets a passing mention*,* yet that’s where many of us come from. I wish our education incorporated real-life rural health issues more extensively.* (Student Nurse 4, Rural origin, studying in an urban setting)

Even when rural exposure was described as professionally meaningful, several participants raised concerns about professional isolation and constrained career development. They framed isolation not only as geographic distance, but as reduced access to mentors, specialist consultation, continuing education, and formal pathways for advancement—factors they perceived as an “opportunity cost” of long-term rural practice.

*The dedication of healthcare workers in Karnali is inspiring. However*,* I can’t ignore the potential for professional isolation. In urban centres*,* you’re constantly surrounded by a network of peers*,* mentors*,* and specialists*,* fostering continuous learning and collaboration. I’m concerned about missing out on mentorship or specialised training. This concern makes me question the long-term sustainability of rural practice.* (Student Nurse 20, Urban origin, studying rurally)

Together, these narratives portray the urban–rural divide as both a material gap (infrastructure, supplies, distance) and a developmental gap (mentorship and career opportunities), shaping how participants evaluated the feasibility and sustainability of rural careers.

#### Theme 5: Gendered barriers: navigating rural practice in Nepal

Participants described gendered and intersectional constraints linked to geography and professional aspirations. While rural service was often framed as meaningful, participants emphasised that the feasibility of rural practice was shaped by gendered risks and social expectations, alongside structural conditions such as housing, transport, and access to professional networks. Concerns ranged from personal safety to reduced opportunities for specialisation. A student nurse studying and residing in the urban setting of Bagmati Province, and not considering rural practice, articulated the pervasive anxieties surrounding personal safety:

*Rural areas come with extra vulnerabilities for women—harassment*,* law enforcement gaps*,* being away from family. It’s not just about providing care; it’s also thinking about your own safety.* (Student Nurse 16, Urban origin, studying in an urban setting)

For some, social isolation was equally pressing. Participants described isolation as both emotional and practical—being distant from family support and peers, and having fewer opportunities to build supportive professional relationships in remote postings.

*There’s a real sense of disconnection from peers if you’re posted far from home. Traditional gender roles can make it harder to integrate or seek support*,* especially in rural settings […]. It can take a toll on your well-being.* (Student Nurse 20, Urban origin, studying rurally)

Others weighed the trade-offs between serving a resource-constrained region and pursuing advanced qualifications. Career progression was frequently described as geographically concentrated, with specialist training and formal advancement pathways perceived to be anchored in major urban centres.

*I’m grateful for KAHS*,* but specialised fields often require advanced programs in bigger cities or abroad. I want to help here*,* yet I also have career ambitions. It’s a tough balance.* (Student Nurse 11, Rural origin, studying rurally)

Despite many expressing altruistic drives, some felt urban centres were unavoidable for sustained professional growth. Participants linked this to access to continuing education, conferences, research opportunities, and networking—resources that they perceived as limited in rural postings.

*Major conferences*,* research*,* networking—it all happens in the city. Missing out on these opportunities might limit my career in the long term. Much as I respect rural service*,* I have to think about my future*,* too.* (Student Nurse 15, Urban origin, studying in an urban setting)

Collectively, these accounts indicate that decisions about rural practice were shaped not only by individual preference but by the intersection of gendered safety concerns, social support structures, and the perceived concentration of professional opportunities in urban settings—factors that directly informed participants’ assessments of whether rural careers were sustainable.

## Discussion

This study reveals a significant difference in the rural-practice intentions of final-year nursing students at the rural-based Karnali Academy of Health Sciences (KAHS) in Nepal’s Karnali Province compared with those of peers enrolled in an urban tertiary institution. Rather than treating this pattern as definitive evidence of a causal “effect,” we interpret it as an indication that rural-embedded education may shape how students *appraise* rural careers, while also illuminating persistent socio-structural constraints that can limit the translation of intention into longer-term rural retention.

Interpreted through Ajzen’s Theory of Planned Behaviour (TPB) [41], participants’ accounts suggest that educational immersion in a remote context may influence intentions by reshaping (i) *attitudes* toward rural practice (e.g., perceived meaningfulness, clinical growth, and community impact), (ii) *subjective norms* (e.g., perceived affirmation and expectations from communities, peers, and educators), and (iii) *perceived behavioural control* (e.g., judgements about whether rural work is feasible given resources, safety, living conditions, and career pathways). Themes describing heightened motivation and purpose (Theme 1) and experiential learning “beyond the books” (Theme 2) align most directly with more favourable attitudinal beliefs and strengthened perceived behavioural control. The relational rewards of community-centred care (Theme 3) appear to reinforce both attitudes and subjective norms by fostering a sense of belonging and professional identity. Conversely, narratives of the urban–rural resource divide (Theme 4) and gendered constraints (Theme 5) highlight barriers that may erode perceived behavioural control—particularly where safety concerns, limited mentorship, and restricted professional development are anticipated.

These mechanisms are consistent with KAHS’s rural-service mandate and rural-embedded education model as described earlier [34, 35], and align with the logic of structural interventions that seek to change the institutional and social conditions shaping workforce decisions, rather than relying solely on individual-level incentives [33, 42]. Structural approaches are increasingly emphasised in LMIC settings where workforce maldistribution is entrenched and inequities are severe [73–75]. In parallel, international “rural pipeline” evidence indicates that locating training in rural contexts and providing sustained rural exposure can increase rural practice outcomes, including among students of urban/metropolitan origin [76, 77]. Research in nursing and allied health fields indicates that rural practice placements have a significant impact on altering professionals’ employment aspirations, often steering them towards careers in rural healthcare settings [78, 79]. Taken together, we therefore use the term “*Karnali Effect*” cautiously as a heuristic label for this constellation of mechanisms, where prolonged rural immersion, community engagement, and exposure to the breadth of rural practice appear to shift TPB-relevant beliefs in ways that support rural career intentions. Importantly, the present study supports *plausibility* rather than causality: intentions were self-reported at a single point in time, and intentions do not always predict subsequent workforce behaviour.

Consistent with this interpretation, the experiential learning and community engagement described by participants resonates with evidence that rural training can support clinical competence, adaptability, and professional identity formation [58, 61, 80]. Participants’ emphasis on relational, community-centred care also aligns with scholarship contrasting transactional urban encounters with longitudinal, trust-based relationships [81], and with literature suggesting that autonomy and breadth of practice can be motivating in under-resourced settings [82, 83]. These convergences strengthen the explanatory value of our findings by situating participant narratives within established pathways through which rural immersion can shape professional values, confidence, and motivation.

At the same time, our findings underscore that strengthened motivation may be undermined by gendered and geographically patterned barriers. Many participants raised concerns about personal safety, harassment, separation from family support systems, and constrained career pathways. These issues align with a “gendered geography of opportunity,” in which infrastructural deficits and sociocultural norms converge to constrain women’s mobility and occupational choices [56, 63, 64]. In addition, participants described anticipated academic/professional isolation (e.g., reduced mentorship, specialist exposure, and continuing professional development), which may compound gendered vulnerability and further weaken perceived behavioural control (TPB) for remote postings. Rural nursing literature similarly identifies professional isolation and constrained development pathways as key drivers of dissatisfaction and turnover [84, 85], reinforcing the importance of analysing retention as a function of both social safety and professional opportunity.

Beyond individual and gendered considerations, participants’ accounts of resource scarcity and urban-oriented curricula highlight broader system determinants of rural workforce distribution [86–89]. Evidence syntheses from LMIC and global contexts indicate that education-based strategies interact with living and working conditions, career pathways, and supportive supervision [90]. Similarly, reviews of rural nurse retention suggest that multi-component approaches, combining education with professional support (e.g., mentoring/supervision), regulation, and enabling infrastructure, are more likely to support sustained retention than single interventions alone [91]. Read together with our themes, these findings suggest that rural-embedded education may be necessary but not sufficient: durable rural retention likely depends on whether the wider rural practice environment enables nurses—particularly women—to feel safe, supported, and able to progress professionally.

Overall, this study contributes context-specific qualitative evidence—grounded in the experiences of final-year female nursing students—that rural-embedded education can reshape beliefs central to rural career intention, including among students from urban origins. By mapping emergent themes to TPB constructs, we clarify plausible mechanisms through which structural investments in rural education may influence workforce aspirations in Nepal and comparable LMIC settings. Equally, our findings highlight that gendered safety concerns and academic/professional isolation remain salient constraints, warranting longitudinal research to examine how these interacting factors shape post-graduation employment trajectories and rural retention.

## Recommendations and future research

Given the qualitative, cross-sectional design and the focus on *intentions* rather than observed post-graduation practice, the recommendations below are presented as context-informed considerations that may be transferable to comparable LMIC settings, rather than prescriptive directives. They are framed in line with structural intervention thinking—where training institutions, working conditions, and professional development opportunities jointly shape workforce distribution.

First, the findings suggest that expanding rural-embedded education pathways may warrant consideration, provided that any expansion is paired with robust evaluation and attention to local feasibility. Evidence from other rural “pipeline” education models indicates that training located in underserved regions can be associated with later rural practice, although causal inferences require careful study designs. In Nepal, this could translate into piloting regionally distributed training hubs or expanding rural placements aligned with national curriculum directions, while explicitly monitoring equity implications and resource demands.

Second, curricular strengthening across both urban and rural institutions may be valuable for reducing urban-centric preparation and building readiness for low-resource practice. This could include structured community-based learning, deliberate preparation for broad-scope practice, and reflective components that support professional identity formation during rural exposure—approaches supported in reviews of rural training impacts [76, 79].

Third, converting rural intentions into retention likely requires packages that address infrastructure and professional support, including safe accommodation, reliable transport, essential supplies, and clear career development pathways—interventions frequently highlighted in rural retention syntheses [21, 89]. To reduce the academic/professional isolation identified in the Results, blended models of mentorship and continuing professional development could be explored, including telementoring/communities-of-practice approaches [92, 93].

Finally, gender-responsive rural workforce supports appear essential in Nepal’s nursing context, where the workforce is exclusively female. Practical measures may include safe housing and transport, mechanisms to prevent and respond to harassment, supportive supervision, and pathways for specialisation that do not require prolonged urban relocation—consistent with wider evidence that gender relations shape safety, mobility, and career progression within health systems [94] and with the gender-related challenges documented in this study.

Future work would benefit from longitudinal designs that follow cohorts from rural and urban institutions into early-career placements to assess (i) the intention–behaviour gap, (ii) retention duration, and (iii) how institutional exposure interacts with posting conditions. Mixed-method or quasi-experimental evaluations could strengthen causal inference by comparing multiple sites and accounting for baseline differences (e.g., rural origin, prior exposure, scholarship/service obligations). In addition, targeted research is needed on gendered safety and professional isolation in remote postings, including how family expectations, supervision quality, and institutional support shape women’s mobility and well-being. Finally, implementation research could test whether digital mentorship/telementoring and locally accessible postgraduate pathways reduce perceived “opportunity costs” of rural practice and improve retention outcomes in.

## Limitations and conclusion

This study was designed as a qualitative descriptive inquiry, prioritising depth of insight and contextual understanding over statistical representativeness. The findings are therefore intended to be interpreted in terms of *transferability*—that is, their relevance to similar rural/remote training and workforce contexts—rather than generalisability. The sample was drawn from two tertiary institutions and comprised only final-year nursing students, which may limit its applicability to other programmes, cadres, or stages of training. In addition, the two sites reflected Nepal’s dual-track nursing education pathways (BNS at the rural site and BSc Nursing at the urban site); differences in prior training exposure and cohort characteristics may have shaped perceptions independently of institutional setting.

The study captured intentions and perceptions at a single point in time. As intentions may evolve following graduation and workplace entry, and may not fully translate into subsequent employment behaviour, causal inferences about workforce retention cannot be made. Finally, although the interview approach aimed to support open discussion, social desirability cannot be ruled out when participants discuss socially valued topics such as rural service.

Using Ajzen’s Theory of Planned Behaviour as an interpretive lens, this study suggests that rural-embedded education may shape nursing students’ rural-practice intentions by influencing beliefs linked to attitudes (perceived meaning and impact of rural work), subjective norms (perceived affirmation and expectations from communities and educators), and perceived behavioural control (perceived feasibility given living, safety, resource, and career conditions). In combination with structural intervention thinking, the findings support the plausibility that an institution anchored in a remote setting and oriented toward community engagement can contribute to a rural workforce pipeline by reshaping how students evaluate rural careers. At the same time, participants’ accounts underscore that intention formation occurs alongside persistent constraints—particularly gendered safety concerns, social and academic/professional isolation, and perceived limits on career development—which may undermine the sustainability of rural practice even among highly motivated graduates. By comparing student perspectives across rural and urban training contexts in Nepal, this study adds context-specific evidence on both the mechanisms that may strengthen rural intentions and the structural conditions that may impede their translation into long-term rural workforce retention.

## Data Availability

The data supporting this study’s findings are available on request from the corresponding author. However, the data is not publicly available due to privacy or ethical restrictions.
